# Biomaterial-Assisted Strategies in Corneal Endothelial Cell Therapy: Toward a Platform-Based Approach

**DOI:** 10.3390/pharmaceutics18060703

**Published:** 2026-06-08

**Authors:** Yura Choi, Mi-Young Jung, Choul Yong Park

**Affiliations:** Department of Ophthalmology, Samsung Medical Center, Sungkyunkwan University School of Medicine, Seoul 06351, Republic of Korea; ychoi.op@gmail.com (Y.C.); myjung202@gmail.com (M.-Y.J.)

**Keywords:** corneal endothelial cell therapy (CECT), ocular drug delivery, controlled cell delivery, ECM-mimicking nanofibers, click chemistry hydrogel

## Abstract

Corneal endothelial dysfunction is a major cause of corneal blindness worldwide. This is primarily due to the limited regenerative capacity of human corneal endothelial cells (CECs) and the global shortage of donor tissues. Corneal endothelial cell therapy (CECT), which involves injecting cultured CECs into the anterior chamber, has emerged as a promising alternative to conventional transplantation. However, its clinical efficacy remains limited by several factors, including rapid cell loss, non-uniform distribution, and insufficient long-term adhesion following injection. Recent advances in biomaterials and regenerative engineering have led to the development of emerging biomaterial-assisted strategies aimed at addressing these challenges. In this review, we provide a mechanistic and translational overview of next-generation CECT, highlighting a range of biomaterial-assisted strategies aimed at improving cell retention, spatial localization, and long-term adhesion following injection. These emerging approaches aim to mitigate key limitations of conventional cell injection therapy, including variability in cell distribution and retention. However, their effectiveness and translational feasibility remain under active investigation. In addition, we analyze recent global patent trends, regulatory frameworks, and market dynamics to highlight emerging opportunities for innovation and development in this field. Although many of these technologies remain at the preclinical or early translational stage, these approaches may provide a promising direction to improve engraftment efficiency, reduce surgical variability, and enable more scalable, minimally invasive treatment options. This review highlights the potential of biomaterial-assisted CECT as a next-generation regenerative strategy and outlines key challenges that must be overcome for successful clinical translation.

## 1. Introduction

Corneal endothelial dysfunction is a leading cause of corneal blindness worldwide, characterized by irreversible loss of corneal transparency due to damage or depletion of corneal endothelial cells (CECs) [1,2]. Because human CECs are non-proliferative in vivo, corneal endothelial disorders such as bullous keratopathy or Fuchs endothelial corneal dystrophy are currently treated through corneal transplantation [2,3]. However, conventional transplantation techniques—including penetrating keratoplasty and posterior lamellar procedures like DMEK or DSAEK—remain limited by donor tissue scarcity, surgical complexity, and postoperative complications [3,4]. In Korea alone, the number of registered patients awaiting corneal transplantation exceeds 2300 annually, while the number of available donor corneas is fewer than 200 per year, resulting in a typical waiting period of over 1800 days [5,6].

This review aims to provide a mechanistic and translational perspective on next-generation corneal endothelial cell therapy (CECT) by examining various biomaterial-assisted strategies with emerging clinical and intellectual property trends. Rather than cataloging technological advances, we focus on how distinct engineering approaches may contribute to addressing the fundamental biological and clinical limitations of current CECT.

**Figure 1:**
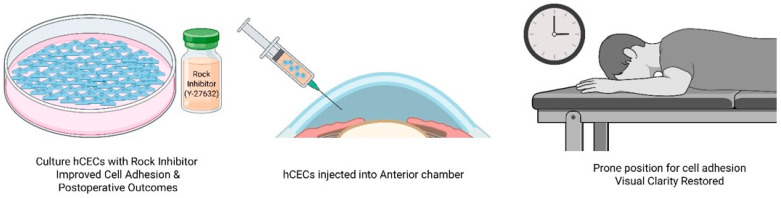
Principle of corneal endothelial cell injection therapy (CECT). Cultured human corneal endothelial cells treated with ROCK inhibitors are injected into the anterior chamber. Patients maintain a prone position for several hours to facilitate adhesion to Descemet’s membrane. Created in BioRender. Jung, M. (2026), https://BioRender.com/j5ic9h2 (accessed on 28 May 2026).

Recent advances in regenerative medicine have enabled the development of CECT, in which cultured CECs are injected into the anterior chamber to restore corneal clarity [7]. Early clinical studies conducted in Japan demonstrated that combining CEC injection with a ROCK inhibitor significantly improves postoperative cell adhesion and visual outcomes, leading to the world’s first regulatory approval of an allogeneic CEC injection product (Vyznova, approved by Pharmaceuticals and Medical Devices Agency in 2023) [7] (Pharmaceuticals and Medical Devices Agency (PMDA), Review report: Vyznova (corneal endothelial cell preparation with ROCK inhibitor Y-27632), Tokyo: PMDA; 2023). This approval has facilitated the development of strategies aimed at addressing current limitations in cell retention, adhesion stability, and long-term functionality [7,8,9,10,11,12,13] (Figure 1).

Despite these advances, current CECT still faces important limitations related to cell retention, spatial localization, and long-term stability following injection, which may contribute to variable engraftment efficiency and inconsistent clinical outcomes.

Building on this clinical foundation, emerging biomaterial-assisted approaches—including scaffold-based systems, biochemical guidance strategies, and material-assisted stabilization methods—have been explored to improve CEC localization, retention, and adhesion to the posterior corneal surface [9,10,14,15,16].

These approaches may help overcome some of the intrinsic limitations of gravity-dependent CECT and support more stable engraftment following transplantation [15,16,17,18,19].

This review provides a comprehensive overview of recent advances in CECT, focusing on representative biomaterial-assisted strategies designed to improve the therapeutic performance and engraftment stability of CECT. These strategies include approaches for:

1. Improving cell expansion and adhesion to enhance cell quality and initial attachment [7,20,21],
2. Enhancing spatial localization through biochemical guidance cues [22,23,24], and
3. Promoting stable fixation using material-assisted systems [15,16,17,18,25].

In addition, we examine global R&D and patent trends, translational challenges, and market perspectives to highlight unmet needs and potential opportunities for next-generation corneal endothelial regenerative therapies.

## 2. Technological Background

### 2.1. Corneal Endothelial Cell Therapy (CECT)

CECT involves the injection of cultured CECs into the anterior chamber, allowing them to attach to the posterior corneal surface and restore endothelial function [7,26]. Compared to conventional keratoplasty, this approach requires fewer donor cells and enables minimally invasive treatment [24,26].

However, the therapeutic efficacy of CECT is highly dependent on post-injection cell behavior, which is governed by both biological and physical constraints within the anterior chamber. In particular, the aqueous humor environment lacks sufficient structural support and biochemical cues to ensure stable cell attachment [10,23].

Consequently, three primary failure modes have been consistently observed: (i) significant early cell loss within hours of injection, (ii) heterogeneous cell coverage due to uncontrolled dispersion, and (iii) detachment or apoptosis of initially adhered cells. These challenges highlight the need for engineered microenvironments that actively regulate cell localization and adhesion (Table 1).

To address these issues, research has shifted toward engineering biomaterial-assisted delivery platforms [10]. This paradigm shift reflects a broader trend in regenerative medicine, where scaffold-based and bioactive cue-driven strategies are increasingly used to guide cell and enhance tissue integration [27].

### 2.2. Ecm Nanofiber Platforms

The Descemet’s membrane-mimicking nanofiber scaffold has emerged as a key technology to support CEC engraftment [15]. Electrospun or electrosprayed ultrathin nanofibers can reproduce the mechanical and topographical properties of the native basement membrane, providing optical transparency and anchoring surfaces for CEC attachment [14,28,29]. Since 2019, multiple academic and industrial research groups have developed high-transparency, mechanically stable ECM-mimicking nanofibers to enhance corneal graft integration, although these approaches remain primarily at the preclinical stage.

**Figure 2:**
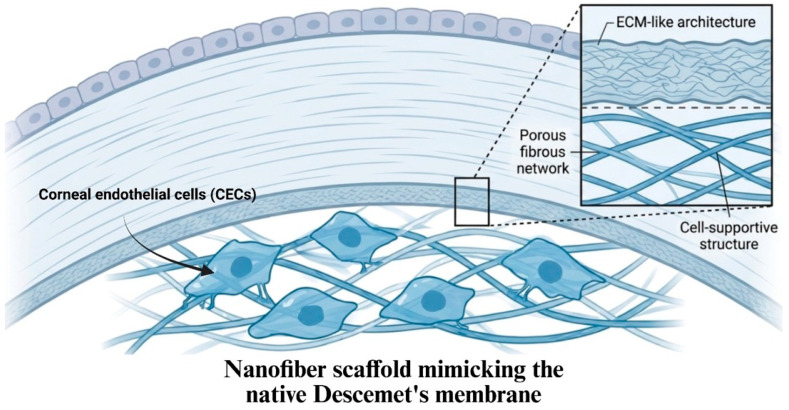
ECM-mimicking nanofiber scaffold for corneal endothelial cell (CEC) engraftment. Created in BioRender. Jung, M., (2026) https://BioRender.com/j5ic9h2 (accessed on 28 May 2026).

From a mechanistic perspective, ECM nanofibers address the initial cell loss problem by increasing physical retention at the posterior corneal surface. Key parameters such as fiber diameter, porosity, and surface chemistry critically influence the cell adhesion strength, spreading behavior, and retention efficiency (Figure 2).

Electrospun nanofibers designed to mimic the native Descemet’s membrane provide a biomimetic substrate that may support initial cell adhesion, spreading, and retention on the posterior corneal surface. The scaffold architecture, including fiber diameter, porosity, and surface properties, may influence optical transparency, mechanical stability, and cellular compatibility.

Reported scaffold properties vary across studies depending on fabrication methods and material composition [13,14,15,18,28,29]. Parameters such as fiber diameter, porosity, mechanical stability, optical transparency, and degradation behavior are considered important factors influencing scaffold performance and potential translational applicability [14,15,18,28,29]. In particular, optimization of the scaffold architecture requires balancing optical clarity, structural stability, and cellular compatibility for corneal applications [14,15,18,28,29] (Table 2).

However, the optimization of ECM-mimicking scaffolds remains challenging because multiple physical properties must be balanced simultaneously. For example, increased scaffold density or fiber thickness may improve mechanical stability and cell adhesion but may also compromise optical transparency and nutrient diffusion [14,18,28]. In addition, variations in fiber diameter, porosity, and degradation kinetics across studies make direct comparison difficult and highlight the need for standardized evaluation criteria for translational application.

### 2.3. Chemokine-Based Cell Guidance & Surface Functionalization

Chemokine-based guidance is an emerging conceptual strategy that may provide directional cues for transplanted CECs, although direct in vivo validation in corneal endothelial injection settings remains limited [22,30,31].

Such approaches have been explored in various regenerative systems and may contribute to improved localization; however, their efficacy in intraocular environments remains to be fully established [22,23,24].

**Figure 3:**
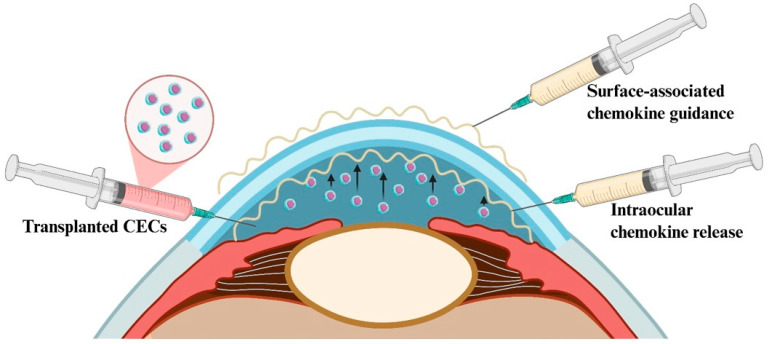
Conceptual illustration of chemokine-guided localization strategies for transplanted corneal endothelial cells (CECs). Intraocular and surface-associated chemokine delivery approaches may generate directional biochemical cues that contribute to the improved spatial localization of transplanted CECs toward the posterior corneal surface. However, stable in vivo chemotactic guidance in corneal endothelial injection settings remains under investigation. Created in BioRender. Jung, M. (2026), https://BioRender.com/j5ic9h2 (accessed on 28 May 2026).

Mechanistically, chemokine gradients may provide directional cues that shift cell behavior from passive sedimentation toward guided migration, although this has not been fully validated in corneal endothelial injection settings This approach may be relevant for addressing uneven cell distribution, a major contributor to incomplete endothelial coverage (Figure 3).

Despite their conceptual potential, chemokine-based guidance systems face several translational challenges in intraocular environments [22,23,24,32,33,34]. Continuous aqueous humor turnover may dilute chemokine gradients and reduce the stability of directional signaling over time [22,32,33,34]. In addition, diffusion limitations, rapid intraocular clearance, and the dynamic fluid environment of the anterior chamber may interfere with sustained spatial guidance of transplanted CECs. Potential inflammatory responses associated with prolonged chemokine exposure should also be carefully considered in future translational studies [32,33,34]. Therefore, further in vivo validation is required to determine whether stable and clinically meaningful chemotactic guidance can be achieved in corneal endothelial injection settings.

**Figure 4:**
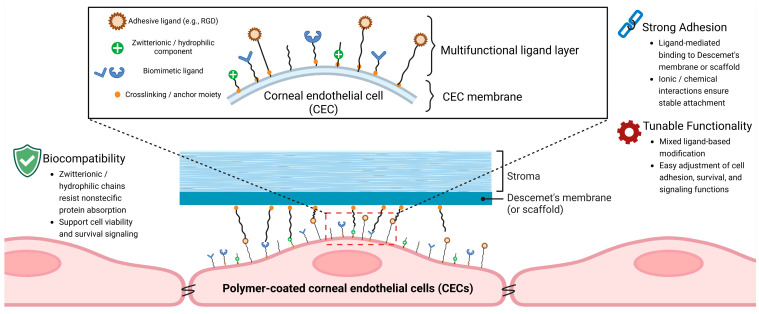
Multifunctional polymer coating for corneal endothelial cells. Design of supramolecular polymer coatings. Functional groups enhance adhesion, biocompatibility, and survival without genetic modification, enabling improved engraftment efficiency. Created in BioRender. Jung, M. (2026), https://BioRender.com/j5ic9h2 (accessed on 28 May 2026).

Nevertheless, chemokine-based systems alone cannot ensure stable retention. Independent of chemotactic strategies, biomaterial-based surface engineering of CECs represents a complementary approach to enhance cell–substrate interactions. These approaches typically involve polymer coating strategies that allow functional ligands to be introduced onto the cell surface without genetic manipulation [7,35,36] (Figure 4).

These coatings can be engineered to enable ionic or covalent interactions with underlying substrates, thereby enhancing adhesion strength. Furthermore, ligand density and composition can be tuned to modulate cell–ECM interactions and improve the survival signaling pathway.

### 2.4. Click Chemistry-Based Hydrogel Fixation

**Figure 5:**
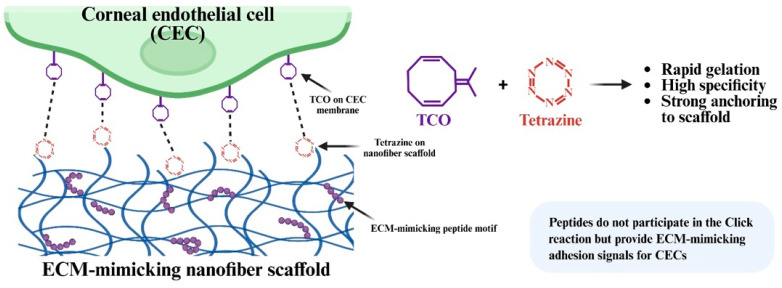
Click chemistry-based nanofiber fixation system. Fabrication of ECM-mimicking nanofiber scaffolds decorated with peptides and tetrazine groups. TCO-engineered corneal endothelial cells undergo bio-orthogonal click reactions, forming stable cell-nanofiber composites for transplantation. Created in BioRender. Jung, M., (2026) https://BioRender.com/j5ic9h2 (accessed on 28 May 2026).

To achieve stable cell adhesion and long-term engraftment, bio-orthogonal chemical reactions are used to rapidly and specifically crosslink ECM nanofibers and hydrogel matrices with CEC membranes [37,38]. This platform offers several advantages: fast gelation under physiological conditions, high reaction specificity, and strong cell–substrate anchoring [39] (Figure 5).

Unlike conventional adhesive systems, click chemistry enables covalent bonding without the need for external catalysts or UV irradiation, making it particularly suitable for intraocular applications. Importantly, reaction kinetics must be carefully controlled, as excessively rapid gelation may hinder uniform cell distribution, whereas slow reactions may fail to prevent early cell loss. In addition, the potential cytotoxicity of reactive groups and their byproducts remain a critical consideration for clinical translation.

Although hydrogel-based stabilization systems provide several advantages for rapid and stable cell anchoring, long-term intraocular biocompatibility remains an important concern [37,38,39,40,41,42]. Residual reactive groups, hydrogel swelling behavior, and potential optical scattering effects may influence corneal transparency and tissue compatibility following transplantation [37,38,42]. Furthermore, regulatory considerations related to chemically modified cellular products may complicate clinical translation and manufacturing standardization [39,40,41].

### 2.5. Conceptual Framework

The convergence of multiple biomaterial-assisted approaches, including optimized cell expansion, biochemical guidance, and material-assisted stabilization, may represent an emerging direction in CECT [23,24]. Rather than relying solely on gravity-dependent settling, these approaches aim to address key limitations associated with cell localization, retention, and long-term stability [10].

**Figure 6:**
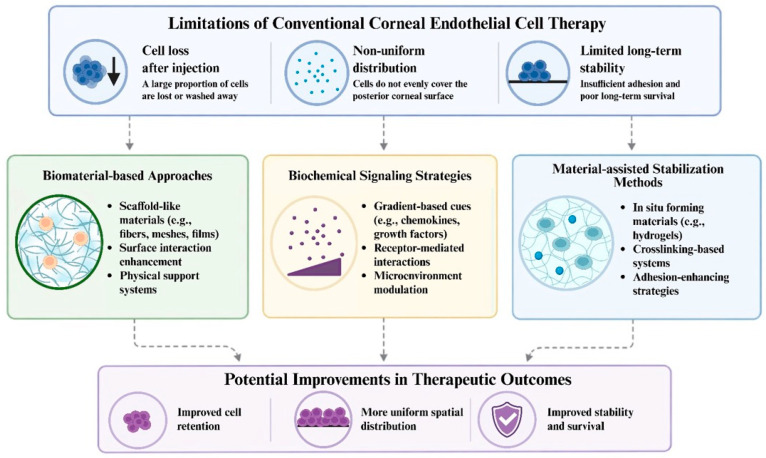
Conceptual framework for biomaterial-assisted corneal endothelial cell therapy (CECT). Key limitations of conventional cell injections, including cell loss, non-uniform distribution, and limited long-term stability, are associated with representative categories of emerging strategies such as biomaterial-based approaches, biochemical signaling methods, and material-assisted stabilization techniques. Created in BioRender. Jung, M., (2026) https://BioRender.com/j5ic9h2 (accessed on 28 May 2026).

For example, scaffold-based strategies may influence early-stage cell behavior, while biochemical guidance mechanisms, such as chemotactic cues, may be associated with cell distribution patterns. In addition, material-assisted stabilization approaches, including hydrogel-based systems, may contribute to cell–substrate interactions and persistence following transplantation. These complementary strategies may help mitigate the sequential limitations of conventional CECT (Figure 6).

Taken together, these approaches may reflect a shift from passive, gravity-dependent processes toward more actively regulated therapeutic strategies, although their effectiveness and clinical applicability remain to be further validated. However, the application of such multi-modal approaches may also introduce additional challenges, including diffusion limitations of signaling molecules within hydrogel matrices, potential interactions between surface modification and stabilization systems, and the need to maintain optical transparency and intraocular safety.

## 3. Patent Landscape and Global R&D Trend

Over the past decade, patent filings in CEC therapy have shifted from basic culture techniques and ROCK inhibition toward biomaterial-assisted delivery systems [10,23,24]. Early-stage patents primarily focused on cell isolation, expansion, and pharmacological enhancement, whereas more recent filings increasingly target scaffold engineering, surface functionalization, and hydrogel-based fixation strategies (Table 3).

### 3.1. Geographic Distribution and Temporal Trends

Patent activity in this field increased steadily until approximately 2017, followed by a gradual decline in overall filings. This trend may reflect a transition from exploratory research toward more clinically oriented development, where fewer but more specialized and translationally relevant patents have emerged. Geographically, the United States, Japan, and Korea represent the primary centers of patent activity [43,44]. The United States maintains leadership in biomaterials and regenerative platforms, while Japan demonstrates strong continuity in clinically driven innovations, particularly in CEC injection therapy [45,46]. Korea has shown a marked increase in filings since 2020, reflecting increased national investment in advanced regenerative medicine technology [44].

Notably, Japan’s sustained patent activity may be associated with its regulatory environment, which enables the accelerated clinical translation of cell-based therapies, as exemplified by the early approval of CEC injection products.

### 3.2. Leading Applicants and IP Strategies

Top applicants include The Regents of the University of California, Kyoto Prefectural University of Medicine, LG Household & Health Care, and Advanced Cell Technology [24,47].

A key observation is the emergence of multi-layered intellectual property strategies, particularly in Japan, where sequential filings encompass cell sourcing, delivery methods, and adjunctive pharmacological modulation (e.g., ROCK inhibitors) [23]. Such strategies may enable the construction of strong patent barriers around a single therapeutic concept.

In contrast, many biomaterial-related patents remain fragmented across scaffold design, hydrogel formulation, and surface modification, indicating an opportunity for integrated platform-level intellectual property.

### 3.3. IP Gap Identification

Analysis of recent patent trends suggests that cell adhesion enhancements and scaffold design remain relatively underexplored domains. These gaps likely reflect both technical and translational challenges rather than a lack of innovation.

For example, achieving rapid bio-orthogonal reactions under intraocular conditions requires strict control over reaction kinetics, biocompatibility, and optical transparency, which complicates patentable implementations.

Importantly, the integration of chemokine-mediated guidance with click chemistry-based fixation remains relatively underexplored, suggesting potential opportunity for developing platform-level intellectual property with translational relevance.

## 4. Market and Regulatory Trends

**Figure 7:**
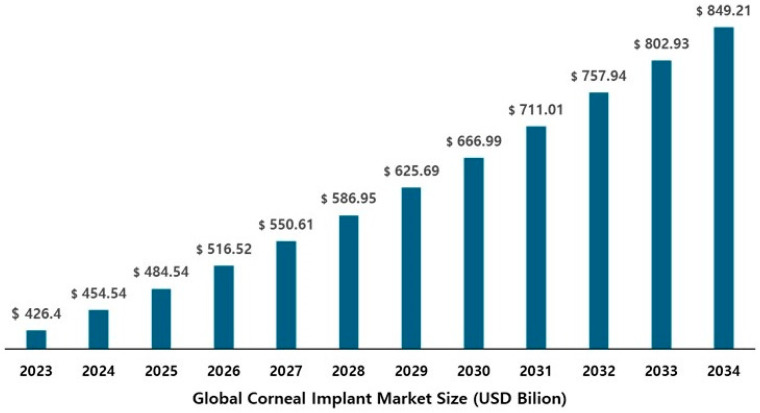
Global market projection for corneal implants and regenerative therapies (2023–2034). Projected growth of the global market from USD 4.26 billion in 2023 to USD 8.49 billion in 2034, driven by demographic trends, donor scarcity, and technological innovation.

The global market for corneal implants and endothelial regenerative therapies is currently undergoing a period of rapid transformation. The market size was valued at approximately USD 4.26 billion in 2023 and is projected to reach USD 8.49 billion by 2034, with a compound annual growth rate (CAGR) of approximately 6.4% [4]. This growth is driven not only by increasing disease prevalence, but also by structural limitations of donor-based transplantation systems, which may be insufficient to meet rising clinical demand (Figure 7).

One of the most significant market drivers is the aging of the global population. Age-related endothelial diseases, including Fuchs endothelial corneal dystrophy, are becoming more prevalent in regions such as North America, Europe, and East Asia. Furthermore, iatrogenic endothelial damage resulting from cataract surgery continues to increase as cataract procedures remain among the most frequently performed surgeries worldwide. In parallel, the supply of donor corneas has stagnated in many regions, with limited availability and unequal distribution creating bottlenecks in conventional transplant systems. This gap between clinical demand and donor supply has become a key factor accelerating the adoption of alternative technologies such as endothelial cell injection therapy and bioengineered corneal substitutes.

In terms of regional market structure, North America currently accounts for the largest share of the global corneal implant and cell therapy market, driven by a well-established healthcare infrastructure, early regulatory pathways for regenerative products, and strong private investment. Europe follows closely, particularly countries such as Germany, the Netherlands, and the United Kingdom, where clinical research on minimally invasive corneal procedures is active. The Asia–Pacific region, however, is projected to experience the fastest growth over the next decade. Japan has already set a regulatory precedent with the approval of Vyznova by the Pharmaceuticals and Medical Devices Agency (PMDA), and South Korea is emerging as a strategic hub for biomaterials and regenerative therapy development, backed by national funding programs. China, with its rapidly growing ophthalmic device and biologics sectors, is expected to expand investment in domestic R&D and manufacturing capacity [12,32].

The industry landscape is also evolving. Historically dominated by corneal transplant and tissue banking organizations, the field is now seeing the emergence of biotechnology and pharmaceutical companies specializing in ocular regenerative medicine. Several global players, including Aerie Pharmaceuticals, CorNeat Vision, and Senju Pharmaceutical Co., Ltd., have initiated or announced programs in corneal regeneration, endothelial cell expansion, or scaffold development. Concurrently, academic institutions and hospital-based R&D centers are driving innovation in early-stage technologies such as ECM nanofiber scaffolds and chemokine-guided delivery platforms, creating fertile ground for future public–private partnerships [11,19,25].

Another critical aspect of this market evolution is the increasing volume of venture capital and public funding directed toward regenerative ophthalmology. In the United States and Europe, early-stage companies have benefited from ophthalmology-focused funds and clinical translation programs that support both IND-enabling studies and early clinical trials. In Asia, particularly in Japan and Korea, government-led translational programs have accelerated the development of clinical-grade manufacturing processes and GMP infrastructure. These funding patterns indicate not only growing confidence in the commercial viability of corneal cell therapy but also a strategic interest in reducing healthcare costs associated with traditional transplantation [40].

From a health economics perspective, regenerative therapies offer a compelling value proposition. Traditional keratoplasty involves not only surgery and donor procurement but also lifelong follow-up, immunosuppressive therapy, and the risk of graft failure or rejection. In contrast, CEC injection therapy offers a minimally invasive, potentially repeatable, and donor-sparing alternative. Economic modeling studies suggest that reducing dependency on donor corneas could result in significant cost savings for healthcare systems, particularly in countries with aging populations and high surgical volumes. This cost-effectiveness, combined with improved patient recovery profiles, is likely to further stimulate adoption.

Finally, regulatory trends are aligning with these market dynamics. Japan’s PMDA conditional approval system has already demonstrated a pathway for bringing allogeneic cell therapies to market. In the U.S., the Food and Drug Administration (FDA) has expanded its RMAT (Regenerative Medicine Advanced Therapy) designation to include ocular cell therapies, providing accelerated review timelines and enhanced guidance for clinical development. Korea’s “Advanced Regenerative Medicine and Advanced Biopharmaceuticals Act” establishes a flexible regulatory structure that supports investigator-initiated trials, compassionate use, and fast-track pathways. These frameworks—ranging from approval systems to designation pathways and national regulatory acts—collectively contribute to an environment that may facilitate early market entry for novel CEC therapies, particularly those with well-characterized manufacturing and safety data [7,11,41,42].

In summary, the corneal implant and regenerative therapy market is shifting from a donor-dependent, surgically intensive model toward a biomaterial- and cell-based therapeutic ecosystem. Demographic pressures, unmet clinical needs, strong R&D investment, and converging regulatory pathways are positioning CEC therapy—and its associated enabling technologies such as ECM scaffolds and click chemistry hydrogels—as a central pillar of the next generation of ophthalmic care [17,18].

While current approvals such as Vyznova represent product-specific innovations, biomaterial-assisted CECT platforms may provide a modular and extensible IP strategy capable of supporting multiple indications, formulations, and delivery configurations under a unified patent framework.

## 5. Future Perspectives and Roadmap

The future of corneal endothelial cell therapy will be shaped by the integration of cell biology, biomaterials engineering, regulatory science, and manufacturing innovation. One of the key steps in this trajectory is the development of Xeno-free and GMP-compliant culture systems capable of generating a stable, clinically relevant supply of high-quality corneal endothelial cells. The gradual replacement of animal-derived serum components with human platelet lysate or platelet-rich plasma formulations is likely to play an important role in meeting the regulatory and clinical requirements, enabling more standardized and reproducible expansion protocols. This shift not only aligns with global regulatory expectations, but also facilitates the creation of scalable production processes suitable for clinical-grade therapy [7,33,40].

In parallel, advancements in targeted delivery systems based on chemotactic gradients may help address a persistent limitation of current injection methods: the uncontrolled dispersion of transplanted cells. By establishing localized chemokine fields, it may be possible to guide cells toward the posterior corneal surface with greater precision, enhancing uniform distribution and retention. When combined with surface functionalization using PEG and cyclodextrin-based polymers, these systems can further increase adhesive strength, promote survival in the early post-transplantation period, and improve the consistency of clinical outcomes [13,19].

A complementary dimension of this roadmap lies in the optimization of hydrogel fixation systems using click chemistry. The unique properties of TCO–tetrazine bio-orthogonal reactions—including rapid gelation under physiological conditions and high reaction specificity—make them suitable for anchoring CECs to ECM-mimicking nanofiber scaffolds. When used in conjunction with other biomaterial-based systems, these approaches may contribute to creating a more supportive microenvironment, although further validation is required. Such approaches may be associated with improved engraftment outcomes [13,17,18,34].

Beyond the technological aspects, ensuring quality control and scalability will be important for clinical translation and commercialization. This involves the establishment of robust cryopreservation protocols, standardized shipping and storage systems, and consistent batch quality control criteria. GMP scale-up strategies incorporating closed-system processing and automated manufacturing are likely to contribute to making these therapies both accessible and economically viable [40].

On the intellectual property and clinical development fronts, targeted patenting strategies focusing on the intersection of chemotaxis and click-chemistry scaffolding may allow innovators to secure strong positions in a rapidly evolving market. Preclinical proof-of-concept studies using appropriate animal models, such as rabbit and porcine eyes, may provide early validation and facilitate proactive engagement with regulatory agencies. Once this groundwork has been established, multinational clinical trials and strategic technology licensing may accelerate global adoption and clinical integration of these biomaterial-enhanced cell therapies.

Taken together, these converging strategies have the potential to redefine how corneal endothelial disorders are treated. By increasing the efficiency of donor cornea utilization, enabling more reliable engraftment, and reducing the risks associated with traditional transplantation, the field may move toward a future where minimally invasive, durable, and scalable CEC therapies could become standard clinical practice worldwide.

Future patent strategies may prioritize interface claims bridging chemotactic cell guidance and click-chemistry-based fixation, rather than isolated material compositions, to establish more robust platform-level protection.

## 6. Conclusions

Corneal endothelial cell therapy (CECT) is emerging as a next-generation regenerative medicine platform designed to overcome the structural limitations of conventional corneal transplantation and reduce dependence on donor tissue. However, its clinical efficacy remains constrained by key challenges, including early cell loss, non-uniform distribution, and insufficient long-term stability following injection.

Biomaterial-assisted strategies, including scaffold-based systems, biochemical guidance approaches, and material-assisted stabilization methods, may provide solutions to these limitations [7,11,17,18,19,25]. ECM-mimicking nanofibers may support initial cell retention, while chemokine-mediated guidance approaches may contribute to improved spatial localization and the distribution of transplanted cells. In addition, hydrogel-based or material-assisted stabilization strategies may enhance cell adhesion and persistence following transplantation. These approaches may collectively contribute to improved engraftment efficiency, spatial distribution, and functional persistence following transplantation [17,18,19,25].

Despite these advances, several challenges remain, including the need to balance optical transparency, biocompatibility, and intraocular safety, as well as the requirement for robust long-term clinical validation.

In conclusion, the convergence of biomaterial-assisted strategies may represent a promising approach to addressing key limitations of current CECT in corneal regenerative medicine. Taken together, these approaches may provide a promising direction for improving the therapeutic performance of CECT, although further validation is required to establish their clinical applicability.

## Figures and Tables

**Table 1:** Representative challenges and emerging biomaterial-assisted approaches in corneal endothelial cell therapy (CECT).

Clinical Challenge	Conventional CECT	Biomaterial-Assisted Approaches
**Cell loss after injection**	Gravity-dependent settling	Scaffold-based or material-assisted retention strategies
**Non-uniform distribution**	Postural control required	Biochemical or physical guidance approaches
**Reoperation risk**	High	Reduced through improved stabilization strategies
**Donor efficiency**	Limited	Expanded through cell amplification or efficient utilization strategies

**Table 2:** Representative physical and translational considerations of ECM-mimicking nanofiber scaffolds for corneal endothelial cell therapy (CECT).

Parameter	Representative Findings	Translational Consideration	References
**Fiber diameter/nanofiber architecture**	Electrospun nanofiber scaffolds with submicron-scale fiber structures have been widely used to mimic the topography of Descemet’s membrane	Fiber architecture may influence cell adhesion, spreading, and scaffold transparency	[14,15,18,28,29]
**Mechanical stability**	Polymer composition influences scaffold flexibility and structural integrity	Mechanical properties must be balanced to maintain surgical handling and intraocular stability	[13,15,18,29]
**Optical transparency**	Transparent or semi-transparent nanofiber membranes have been developed for corneal applications; one gelatin nanofiber membrane demonstrated ~80% transparency relative to glass	Optical clarity is essential for clinical translation and postoperative visual function	[14,18,28]
**Degradation behavior**	Scaffold degradation properties vary depending on polymer composition and crosslinking methods	Degradation kinetics should support temporary cell retention without prolonged optical interference	[13,14,18,29]
**Cell adhesion/compatibility**	ECM-mimicking nanofiber scaffolds improve CEC attachment and support the expression of functional markers such as ZO-1 and Na^+^/K^+^ATPase	Stable early adhesion and maintenance of endothelial phenotype are important for engraftment	[14,15,18]

**Table 3:** Representative patents related to biomaterial-assisted strategies and delivery platforms in corneal endothelial cell therapy (CECT).

No	Applicant	Title	Patent No.	Year	Category
1	Kyoto Prefectural Public University Corporation	Human functional corneal endothelial cell and application thereof	US 16/078002	2017	Cell therapy
2	Gachon University	ECM-based click chemistry hydrogel platform	KR 2023-0026696	2023	Biomaterials
3	Incheon National University	Gelatin-based tissue adhesive hydrogel	KR 2022-0096098	2022	Hydrogel
4	Ocean University of China	Tissue-engineered human corneal endothelium	EP 2412799	2012	Scaffold

## Data Availability

No new data were created or analyzed in this study. Data sharing is not applicable.

## Abbreviations

CECs	Corneal Endothelial Cells
CECT	Corneal Endothelial Cell Therapy
DMEK	Desmet Membrane Endothelial Keratoplasty
DSAEK	Descemet Stripping Automated Endothelial Keratoplasty
ECM	Extracellular Matrix
FDA	Food and Drug Administration
GMP	Good Manufacturing Practice
IP	Intellectual Property
PEG	Poly(ethylene Glycol)
PMDA	Pharmaceuticals and Medical Devices Agency
RMAT	Regenerative Medicine Advanced Therapy
ROCK	Rho-Associated Protein Kinase
TCO	Trans-Cyclooctene
